# How Hot Are Drosophila Hotspots? Examining Recombination Rate Variation and Associations with Nucleotide Diversity, Divergence, and Maternal Age in *Drosophila pseudoobscura*

**DOI:** 10.1371/journal.pone.0071582

**Published:** 2013-08-13

**Authors:** Brenda Manzano-Winkler, Suzanne E. McGaugh, Mohamed A. F. Noor

**Affiliations:** 1 Biology Department, Duke University, Durham, North Carolina, United States of America; 2 The Genome Institute, Washington University, St. Louis, Missouri, United States of America; University of Iceland, Iceland

## Abstract

Fine scale meiotic recombination maps have uncovered a large amount of variation in crossover rate across the genomes of many species, and such variation in mammalian and yeast genomes is concentrated to <5****kb regions of highly elevated recombination rates (10–100x the background rate) called “hotspots.” *Drosophila* exhibit substantial recombination rate heterogeneity across their genome, but evidence for these highly-localized hotspots is lacking. We assayed recombination across a 40****Kb region of *Drosophila pseudoobscura* chromosome 2, with one 20****kb interval assayed every 5****Kb and the adjacent 20****kb interval bisected into 10****kb pieces. We found that recombination events across the 40****kb stretch were relatively evenly distributed across each of the 5****kb and 10****kb intervals, rather than concentrated in a single 5****kb region. This, in combination with other recent work, indicates that the recombination landscape of *Drosophila* may differ from the punctate recombination pattern observed in many mammals and yeast. Additionally, we found no correlation of average pairwise nucleotide diversity and divergence with recombination rate across the 20****kb intervals, nor any effect of maternal age in weeks on recombination rate in our sample.

## Introduction

Recombination plays a major role in determining the effects of natural selection on the genome [1], [2], [3], and unaccounted variation in recombination rate may complicate genetic mapping studies [4] or evolutionary inferences from population genetic data [5]. Even when recombination rates are known, accounting for variation in recombination rate is often not straightforward. One confounding factor is that the scale at which recombination is measured has been different across studies [6]. Genetic or evolutionary interpretations are thereby challenging, especially since recombination rate is often conserved between closely related taxa at broad scales and highly divergent at fine-scales [7], [8], [9], [10], and certain genomic features are correlated with recombination rate at some scales but not others [11], [12], [13]. In response, there has been a recent push to more precisely measure recombination rates on finer scales, with the understanding that broader scale measures can always be obtained from interpolating across multiple fine scale intervals.

Recent studies have found that the genomes of many species exhibit extensive variation in fine-scale recombination rate [10], [13], [14], [15], [16], [17], [18], [19], which may not be apparent when recombination is studied in megabase-sized or larger intervals. For instance, in humans, mice, and chimps, crossover activity is concentrated in 1–2****kb stretches surrounded by large regions of essentially no recombination [14], [20], [21], resulting in 80% of recombination events localized to just 10–20% of sequence in primates [10], [14] and mice [22]. Much of this fine-scale heterogeneity in crossover rate in humans and mice is mediated by the binding of the protein PRDM9 to specific DNA sequence motifs across the genome. In organisms that lack a functional copy of *Prdm9*, the general finding is that recombination is not as punctate [16]. To distinguish between these highly punctate regions and regions with elevated recombination rates which are not as tightly focused, numerous terms have been used to describe the variation in crossover rate within genomes. The terms “hotspots” and “coldspots” are used to describe highly localized recombination events, with extreme or statistically significant recombination differences relative to background [14], [23], [24]. Since their initial use, other features have come to be associated with a stereotypical hot or cold spot [14]. For recombination measures across broader regions, areas of high recombination have been called “jungles” [25], [26], “peaks” [15], or “windows” [12] which are often defined as ∼1 Mb chunks of the genome with the top 10% recombination rates [26]. Despite these differences in nomenclature, it is unknown if jungles, peaks, and windows, actually harbour stereotypical hotspots.

One key taxonomic group for which this issue remains unresolved is *Drosophila*, despite a century of history generating linkage maps in *D. melanogaster.* Because linkage disequilibrium erodes quickly in *Drosophila* [27], it was relatively surprising when fine-scale examination revealed significant crossover rate variation [9], [13], [15], [28], [29], [30]. Recent studies have looked for mammalian-like recombination hotspots in *D. melanogaster*: one used linkage disequilibrium (LD) across the whole genome and found approximately 10 hotspots [defined as a ten-fold increase in recombination relative to adjacent intervals: ref 9], and others used next-generation sequencing to genotype offspring from a cross and directly identify variation in crossover rate [13], [29]. These studies seem to suggest that *D. melanogaster* has “a softer, more probabilistic and less discrete, [recombinational] landscape” than humans or yeast [29]; and zinc-finger motifs, like *Prdm9*, have little explanatory power in *Drosophila* recombination [31]. Further studies precisely localizing recombination events in other Drosophila species can identify whether this softer recombination landscape is unique to *D. melanogaster*. *D. melanogaster* also exhibits maternal-age-effects on crossover frequency [32], varying among regions of the genome, and possibly resulting from changes in the frequency of double-exchange tetrads [33]. This age effect, too, merits investigation in other Drosophila species.

*Drosophila pseudoobscura* offers some key advantages over *D. melanogaster* as a system in which to look for fine-scale crossover rate heterogeneity. First, crossover rates are on average higher in *D. pseudoobscura* than *D. melanogaster* [34], providing more power for quantifying crossover rate with a finite number of progeny. Second, significant crossover rate heterogeneity has been identified at both the ∼150-kb scale and ∼20-kb scale in *D. pseudoobscura* [17], and over ten-thousand progeny used for studying crossover rate at the latter scale are available for further genotyping. Finally, *D. melanogaster* appears to harbour very extensive among-strain variation in local recombination rates [29] whereas our prior examination in *D. pseudoobscura* failed to identify such variation at the same scale [17].

Here, we measured recombination rate at the 5–10****kb scale in a cross of *D. pseudoobscura* inbred lines to test whether Drosophila exhibit true “hotspots”. Specifically, we dissected a 40****kb region of chromosome 2 in *Drosophila pseudoobscura*, which was previously shown to have high levels of fine-scale recombination rate heterogeneity, into 5****kb and 10****kb intervals to examine recombination rate on an extremely fine-scale. We also test for an association of fine-scale recombination rate with DNA sequence diversity or divergence between-species and examine the influence of maternal age (crudely quantified by weeks of adulthood) in affecting fine-scale crossover rate.

## Methods

### Crossover Maps: Marker Development and Recombination Map Construction

Recombination was measured intensively for three ∼100–125****kb regions on chromosome 2 (referred to here as 6 Mb, 17 Mb, and 21 Mb) by placing a marker every 20****kb [for additional details see 17]. Markers were designed by viewing alignments of Flagstaff14 and Flagstaff16 to the reference *D. pseudoobscura* genome as sorted bam files in Integrative Genomics Viewer (IGV) v. 1.4.04 [35] to identify indels between the two lines. These regions spanned positions 6.003 Mb–6.108 Mb (6 markers, 5 intervals, average interval size of 20.280****kb), 17.534 Mb–17.660 Mb (7 markers, 6 intervals, average interval size of 20.878****kb), and 21.438 Mb–21.537 Mb (6 markers, 5 intervals, average interval size of 19.870 kb) on chromosome 2. We aligned 76bp, 9****kb mate-paired Illumina reads to the reference genome with bwa, and confirmed that insert sizes were the expected size for multiple read pairs. Thus, we ensured the distance between markers was that predicted from the published genome sequence assembly [36]. Figure S1 shows the recombination rates identified by McGaugh et al. [17] from these ∼100–125****kb regions.

New recombination analyses presented here focused on the first 40****kb of the 17 Mb region discussed above. Markers were designed by viewing alignments of genome resequence data (described in [17]) from the inbred lines Flagstaff14 (collected from Flagstaff, AZ in 1997) and Flagstaff16 (Flagstaff, AZ, 1997) to the reference *D. pseudoobscura* genome as sorted bam files in IGV [35] to identify SNPs between the two lines. Line-specific primers using these SNPs were developed to produce differentially sized products for the two lines which could be visualized on the LICOR 4300. The first 20****Kb region (hereafter “17.1”) spans *D. pseudoobscura* assembly positions 17.534 Mb to 17.555 Mb and had a reported recombination rate of 21.3 cM/Mb [17]. This region was assayed with 5 markers (17.1, 17.1b, 17.1c, 17.1d, 17.2), spaced roughly 5****kb apart (four total intervals of 5****kb each). The second 20****Kb region (“17.2”) extended from 17.555 Mb to 17.575 Mb and had a reported recombination rate of 6.9****cM/Mb [17]. The 17.2 window was bisected with one marker (17.2b), resulting in two windows spanning 10****kb each.

To measure recombination rate over a single generation in these windows, *D. pseudoobscura* females from the Flagstaff 16 inbred line were crossed with males of the Flagstaff 14 inbred line. F1 females were backcrossed to Flagstaff 16 inbred males. The timing of the crosses was noted in that Flagstaff 16 virgin female flies were kept in isolation for 6–7 days and transferred to a vial with 2–3 males on the 6^th^ or 7^th^ day. On the 10^th^ day of life, the females and their mates were transferred into a new vial. After 7 additional days, all flies were removed from this vial. For the F1s, females were kept in isolation for 6–7 days and then transferred to a vial with 2–3 Flagstaff 16 males. Flies were kept for 9–10 days and flipped into a new vial at which point they were kept for 7 additional days and then discarded. More than 10,000 backcross progeny were collected in 96-well plates and placed in a −20°C freezer. DNA was extracted from flies by adding 63.5ul squish buffer (10 mM Tris-HCl (pH 8.2), 1 mM EDTA, 25 mM NaCl)+1.3 proteinase K [37], placing a Zirconium bead in each well, and sealing the plate with packing tape for 7 minutes at 23°C. A Qiagen TissueLyser II was used to shake the plates for 45 seconds.

While slight modifications were used in some cases, the PCR reagents generally consisted of 0.5 uM forward primer+M13 tag, 0.5 uM reverse primer, 0.1 uM 700IRD or 800IRD-labeled M13 tag, 1.5 mM MgCl_2_, 1X buffer, 0.2 mM dNTPs, and 1U Taq polymerase in a 10 uL reaction volume. Generally, the PCR program included an initial denaturing step of 94°C for 60 sec, three touch-down cycles of 94°C- 58°C- 72°C for 30 sec each, followed by 31 main cycles of 94°C- 56°C- 72°C for 30 sec each. Products were visualized on a 5% polyacrylamide gel using a LICOR 4300. A total of 10,160 backcross progeny were assayed, and 95% confidence intervals for recombination rate for each recombination interval were calculated by permutation [28], [30].

Computational and statistical methods for obtaining and assessing significance of recombination rate relative to average pairwise nucleotide diversity (π) and divergence were identical to those described in McGaugh et al. [17]. Because window sizes of the map were ∼5–10****kb, few bases were eligible for four-fold degenerate synonymous site diversity and divergence analysis. Therefore, we used intergenic regions for analysis, but include the four-fold degenerate measures in the supplementary materials. To account for the non-independence of the intervals within the same region (i.e. 6 Mb, 17 Mb, 21 Mb regions) a binomial generalized linear mixed model (GLMM) was implemented with “region” as a random effect.

We used a rare events logistic regression in the R package Zelig to assess the effect of the age of the F1 female in creating recombinant backcross progeny. A rare events logistic regression is similar to a standard logistic regression, but the former accounts for the rarity of the event in question (e.g. recombination). Grandmother’s age and maternal age were each binned into two groups “Young” or “Old.” “Young” corresponds roughly to post-eclosion adult day 7–10 of life and day 7–17 of life for grandmother (the parental Flagstaff 16 female) and mother (the F1 female), respectively. “Old” corresponds roughly to adult day 10–17 of life and day 17–24 of life for grandmother and mother, respectively. Two models were run and both contained recombination status of an individual fly (denoted as a binomial variable 1 = recombinant, 0 = non-recombinant) as the response variable. In the first model, the independent variables were grandmother’s age and mother’s age. The second model was identical to the first, except grandmother’s age was removed. Data from all three 120****kb regions (6 Mb, 17 Mb, 21 Mb) were included in the model. While most values for the response variable were “0,” we had a total of 169,565 measures across all individuals for all intervals contained in all three regions.

## Results

### Hot Windows, but no Punctate “Hotspots” in *Drosophila pseudoobscura* 17 Mb Region

**Figure 1:**
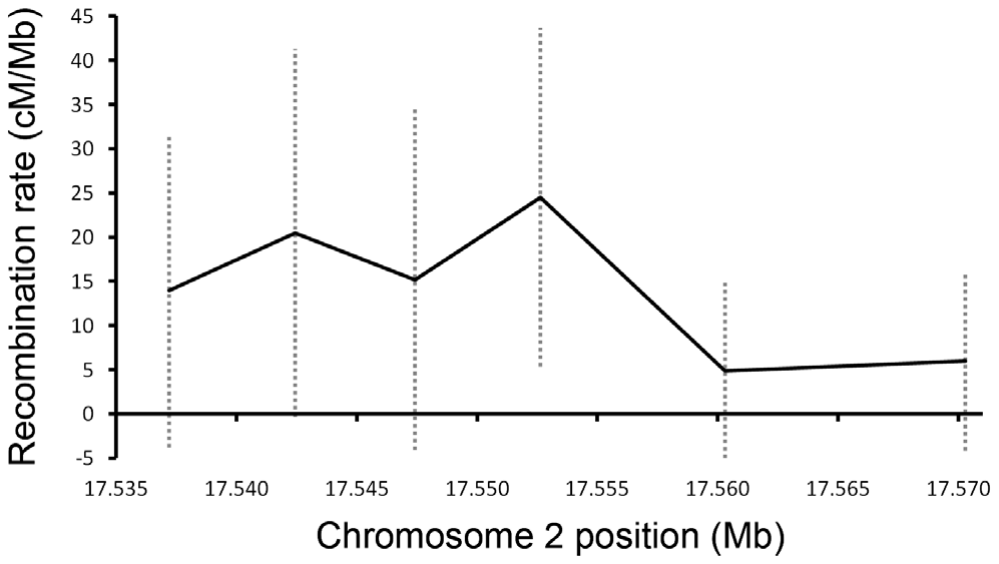
Recombination rate in the 17 Mb region between markers 17.1 and 17.3. Dotted lines are 95% confidence intervals.

Our genotyping results confirmed the elevated recombination rate reported previously [17]. We identified 40 recombinants in the 17.1 window and 11 recombinants at the 17.2 window. The results from the four 5****kb regions revealed a fairly even distribution of crossovers across the entire 20****kb region of elevated recombination (17.1): recombination rates ranged from 14****cM/Mb to 24.5****cM/Mb (Table 1; Figure 1) which all had overlapping 95% confidence intervals. In addition, we examined the adjacent 20****kb window with markers 10****kb apart and found a pattern of even crossover distribution: 4.9****cM/Mb and 6 cM/Mb, which is close to the genome-wide average recombination rate (5****cM/Mb). Hence, recombination was slightly elevated across the entire 20****kb span of the 17.1 window relative to the 17.2 window or the genomewide average, rather than tightly focused into one or two small punctate regions of very high recombination.

**Table 1:** Raw recombination data and 95% confidence intervals.

Interval	Marker1	Marker2	bp	Recombinant Individuals	cM/Mb	95% confidence interval
17.1_17.1b	17534400	17540029	5629	8	13.99	(−3.78, 31.75)
17.1b_17.1c	17540029	17544832	4803	10	20.49	(−0.33, 41.31)
17.1c_17.1d	17544832	17550024	5192	8	15.17	(−4.09, 34.43)
17.1d_17.2	17550024	17555244	5220	13	24.51	(5.35, 43.67)
17.2_17.2b	17555244	17565333	10089	5	4.88	(−5.03, 14.79)
17.2b_17.3	17565333	17575208	9875	6	5.98	(−4.15, 16.11)

“Marker1” and “Marker2” refer to the physical assembly location of the markers flanking each interval. “bp” refers to the size of the interval. Total number of backcross individuals genotyped was 10,160.

### No relationship of Diversity or Divergence with Recombination

**Figure 2:**
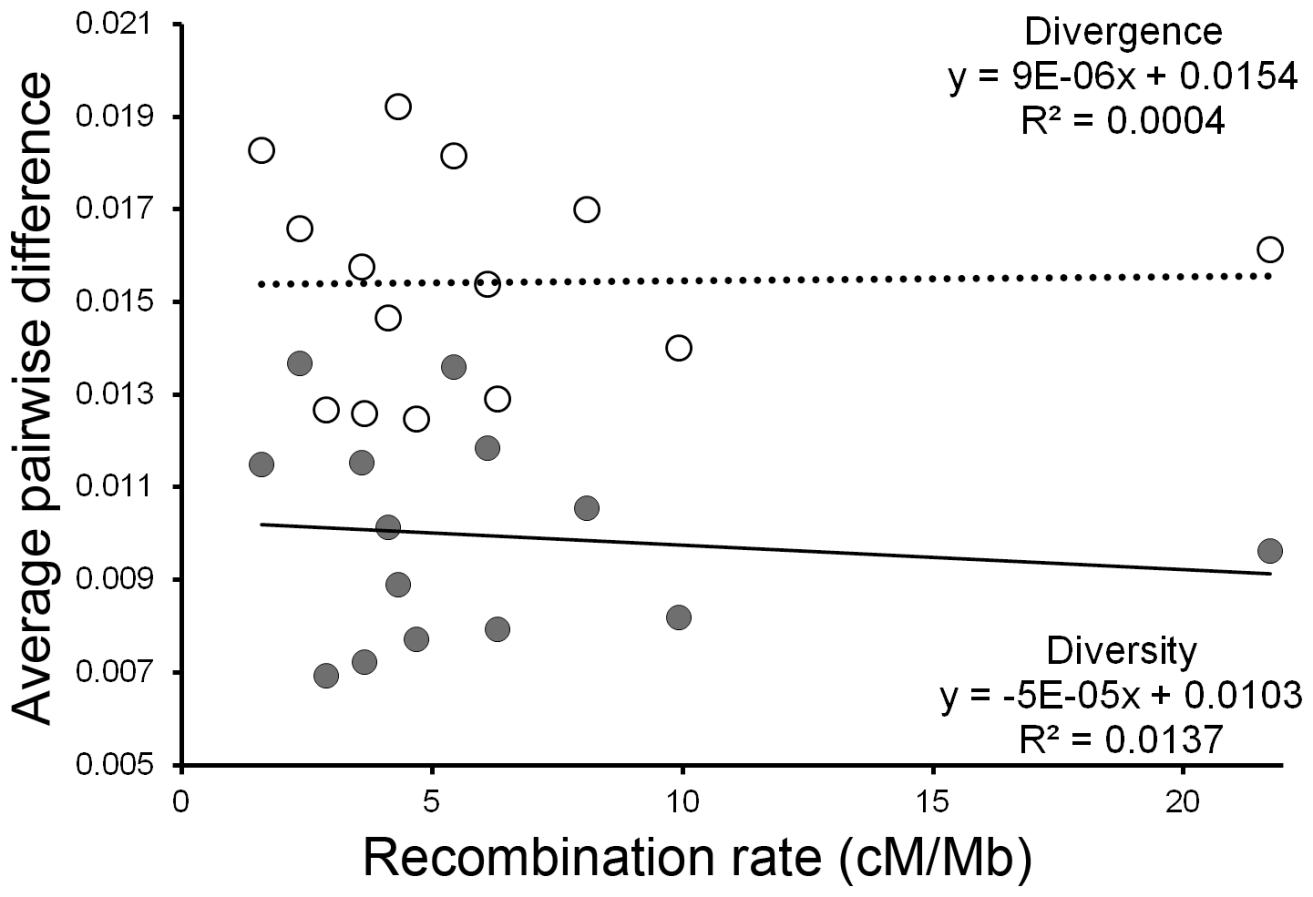
Diversity and divergence in relation to recombination rate for intergenic regions. Kosambi recombination rate relative to diversity within *D. pseudoobscura* (grey circles, t = −0.4088, df = 12, p = 0.6899) and divergence between *D. pseudoobscura*-*D. miranda* (white circles, t = 0.0697, df = 12, p = 0.9456) for the intervals using intergenic bases in the measure of diversity and divergence because there were few fourfold degenerate sites within a single 20****kb window (but see Figure S2).

The recombination-diversity association was examined using 20****kb windows within the three ∼100–125****kb regions (6 Mb, 17 Mb, 21 Mb). We focused on diversity and divergence measures from intergenic regions (see Methods). In contrast to an earlier study that used recombination measures derived from 150****kb windows [17], we found no significant relationship between recombination and either average pairwise nucleotide diversity or divergence. This nonsignificant result was likely not due to a lack of power because the correlation coefficients indicated recombination rate explained very little of the variation in diversity or divergence (Figure 2; R^2^ = 0.0137 for diversity, R^2^ = 0.0004 for divergence) (Figure 2, Table 2). Though the number of eligible SNP and non-SNP bases for the four-fold degenerate sites were few and could give inaccurate results, average pairwise nucleotide diversity and divergence at four-fold degenerate synonymous sites corroborated the results from the intergenic regions (Figure 2; R^2^ = 0.1197 for diversity, R^2^ = 0.0196 for divergence) (Figure S2, Table S1). In short, there was no significant association of recombination with these measures of diversity or divergence in these regions at the scale examined.

**Table 2:** Test for relationship between recombination rate and diversity and divergence for sites in intergenic regions.

Response: Diversity at sites in intergenic regions				
Factor tested	Estimate	Std. error	z-value	p-value
(Intercept)	−2.9	1.667	−1.739	0.082
Mutation	5.082	10.004	0.508	0.611
GC content	−3.937	3.569	−1.103	0.27
Gene density	0.19	0.53	0.359	0.72
**Recombination**	**−0.006**	**0.0075**	**−0.754**	**0.451**
**Response: Divergence at sites in intergenic regions**				
**Factor tested**	**Estimate**	**Std. error**	**z-value**	**p-value**
(Intercept)	−4.914	1.383	−3.553	0.0004*
Mutation	11.08	8.642	1.282	0.1998
GC content	0.726	2.925	0.248	0.8041
Gene density	0.08	0.422	0.189	0.8504
**Recombination**	**0.004**	**0.007**	**0.617**	**0.5372**

Two generalized linear mixed models with binomial distribution. Region (6 Mb, 17 Mb, 21 Mb) was included as a random effect to account for including multiple intervals per region. This analysis only included 20****kb regions. An asterisk indicates significance at α = 0.05. For this analysis, the ‘neutral mutation rate’ was set as the average pairwise *D. lowei*-*D. persimilis* divergence for sites in intergenic regions.

### No relationship of Age of Mother or Grandmother with Recombination

The rare events logistic regression, which accounts for the rarity of the event in question (e.g. recombination), did not provide evidence that grandmother’s or mother’s age had a significant impact on recombination over the regions (6 Mb, 17 Mb, 21 Mb) examined. The conclusion that mother’s age had no relationship with recombination rate was not affected by the inclusion or exclusion of grandmother’s age in the model (absolute z-value <0.501, p-value >0.62, in both models).

## Discussion

While the number of species for which we have detailed recombination maps is growing, many of these maps do not have sufficient resolution to detect or exclude the presence of hyper-localized recombination hotspots [26], [38], [39], [40], [41], [42], [43]. In our study, we dissected a 40****kb region of chromosome 2 in *Drosophila pseudoobscura*, which was previously shown to have high levels of fine-scale recombination rate heterogeneity, into 5****kb and 10****kb intervals to examine recombination rate on an extremely fine-scale. Even when directly measuring single-generation recombination rate on the 5****kb-scale, there was no stereotypical mammalian-like recombination hotspot in this region. While the "gold standard" would be to sequence the regions and pinpoint each individual crossover, the data available strongly suggest an elevated recombination rate across the entire 20****kb window. We also found that the recombination rate never fell below 4****cM/Mb across the 40****kb region examined.

Most areas of human, mouse, chimp, and yeast genomes experience very low recombination rates, and the majority of recombination in those genomes occurs in localized "hotspots" that are spaced tens of kilobases apart, on average [10], [22], [44]. Similarly punctate crossover rate heterogeneity has also been documented genome-wide in *Arabidopsis* [45] and *Medicago* [12]. Many reviews have argued that hotspots of this sort are absent in *Drosophila* because of the rapid and consistent breakdown of linkage disequilibrium with physical distance [27], [46], [47], [48], [49], [50] as well as detailed assays of intragenic recombination within the *rosy* locus which showed that recombination is not restricted to a subset of particular sites or concentrated in one small area within this locus [51], [52], [53]. However, the breakdown of linkage disequilibrium may suggest a higher "basal" rate of recombination rather than an absence of punctate hotspots.

Our results agree with the findings that the *Drosophila* recombination landscape may depart from the classic depiction of hotspots derived from yeast, human, and mouse studies in two ways in particular. We observed a much higher background recombination rate than those reported in humans, mice, and yeast [10], [40], [41], [44]. Secondly, while we did not observe regions of elevated recombination tightly focused into <5****kb stretches, our study confirms that *Drosophila* experience regions of significantly elevated recombination over background. Other recent studies have reported similar findings [13], [17], [28], [29]. For instance, Singh et al. documented recombination rate fluctuations of almost two-orders of magnitude in *Drosophila melanogaster* when measured at a similar scale as examined by the present study [13]. Indeed, across the entire genome of *D. melanogaster*, 10 putative hotspots were found across two separate recombination maps through computational population-based inference (LDhelmet) for estimating recombination rates within a high recombination rate background [9]. Seven of these have a 10-fold increase over background and 1–2****kb width. The 3 other hotspots had widths between 4.1–6.8****kb. As we learn more about the resolution of crossovers in different species, the term "hotspot" should not be limited to the specific features of just a few species, and should not necessarily imply a common mechanistic basis for the elevated recombination rates [10], [54]. Certainly, such elevated rates as seen here fit one of the earliest definitions of recombination hotspots [24].

Many differences between mammals and Drosophila could account for the dissimilar recombination profiles. For example, PRDM9 determines recombination hotspots in many mammals, but Drosophila not only lacks PRDM9 itself but also lacks evidence of a similarly functioning zinc-finger binding protein [31]. The density of genes differs greatly between the two genomes. Additionally, germline methylation levels correlate with regional levels of meiotic recombination in humans, but patterns of methylation are dramatically different (and lower) in Drosophila [55], including *D. pseudoobscura* [56], possibly contributing to differences in patterns of recombination. Many other factors differentiating mammals and Drosophila may also contribute.

We also replicated results of Singh et al. [13] revealing no relationship between recombination rate, divergence, and diversity at such a fine-scale. We doubt the nonsignificant result is due to a lack of power because recombination rate did not explain much of the variation in diversity or divergence (Figure 2; R^2^ = 0.0137 for diversity, R^2^ = 0.0004 for divergence), and was not necessarily even positive in direction. One explanation for our results could be that hitchhiking in *Drosophila* produces effects that are longer in range than 20****kb, and the effects are therefore not evident when zoomed in on such a fine-scale [11], [13]. This explanation remains somewhat tentative or taxon-specific, though, because recombination rates measured at the 1–2****kb scale in *Arabidopsis* and *Medicago* exhibit a positive association with diversity [12], [57].

Finally, crudely measured maternal age appears to have little influence on recombination rate for our dataset. This finding contrasts those from studies that showed increasing maternal age can impact recombination rate, but those impacts are non-linear in *Drosophila* [32], [58], [59]. Likewise, increased maternal age is associated with an overall decrease in recombination rate in humans [60] and *C. elegans* [61], [62], though in both taxa, this effect is associated with a change in the physical distribution of crossovers, as well. One reason we may not have detected an age effect may be because recombination is relatively infrequent when intervals are 20****kb in size, and very large sample sizes or very large effect sizes are required to be able to detect a statistical difference between “Young” and “Old.” Although we cannot rule out this possibility, we do not favour a lack-of-power explanation for our result because our data was sufficient to detect rate differences between regions (6 Mb, 17 Mb, and 21 Mb, results not shown). We suspect that the coarseness of our binning of “Young” and “Old” may have prevented us from detecting some age effects that may actually be present: the effects detected by Redfield [32] showed dramatic differences between females differing in age by only 2 days, and the effects were not strictly linear with age.

In conclusion, our data suggest that crossover distribution and intensity in *D. pseudoobscura* may be different than the punctate, highly elevated crossover hotspots seen in many mammals and yeast. Our findings in *D. pseudoobscura* corroborate results of recent studies in *D. melanogaster* [9], [13]. In addition, we did not find any correlations of recombination rate to diversity, divergence, or maternal age at this fine scale.

## Supporting Information

Figure S1 **Recombination rate over the 100–125 kb regions.** Each window is approximately 20****kb. Approximately 10,000 individuals were scored across each window. A similar figure was presented in [17].
(TIF)

Figure S2 **Diversity and divergence in relation to recombination rate for four-fold degenerate sites.** Kosambi recombination rate relative to diversity within *D. pseudoobscura* (grey circles, t = −1.3297, df = 13, p = 0.2067) and divergence between *D. pseudoobscura*-*D. miranda* (white circles, t = −0.5098, df = 13, p = 0.6187) for the intervals using four fold degenerate bases in the measure of diversity and divergence. The number of data points was governed by the availability of sites for diversity and divergence measures in each recombination interval; thus, the number of data points in this figure is different from the analogous Figure2 which used data from bases in intergenic regions. Divergence: y = −0.0003x+0.0348, R^2^ = 0.0196; Diversity: y = −0.0005x+0.0252, R^2^ = 0.1197.
(TIF)

Table S1 **Test for relationship between recombination rate and diversity and divergence at four-fold degenerate sites.** Two generalized linear mixed models with binomial distribution. Region (6 Mb, 17 Mb, 21 Mb) was included as a random effect to account for including multiple intervals per region. This analysis only included 20****kb regions. For this analysis, the ‘neutral mutation rate’ was set as the average pairwise *D. lowei*-*D. persimilis* divergence at four-fold degenerate sites.
(DOCX)
