# Supplementary figures and images for: How Hot Are Drosophila Hotspots? Examining Recombination Rate Variation and Associations with Nucleotide Diversity, Divergence, and Maternal Age in Drosophila pseudoobscura
Source: PLoS One. 2013 Aug 13;8(8):e71582. doi: 10.1371/journal.pone.0071582 (PMC3742509; doi:10.1371/journal.pone.0071582)

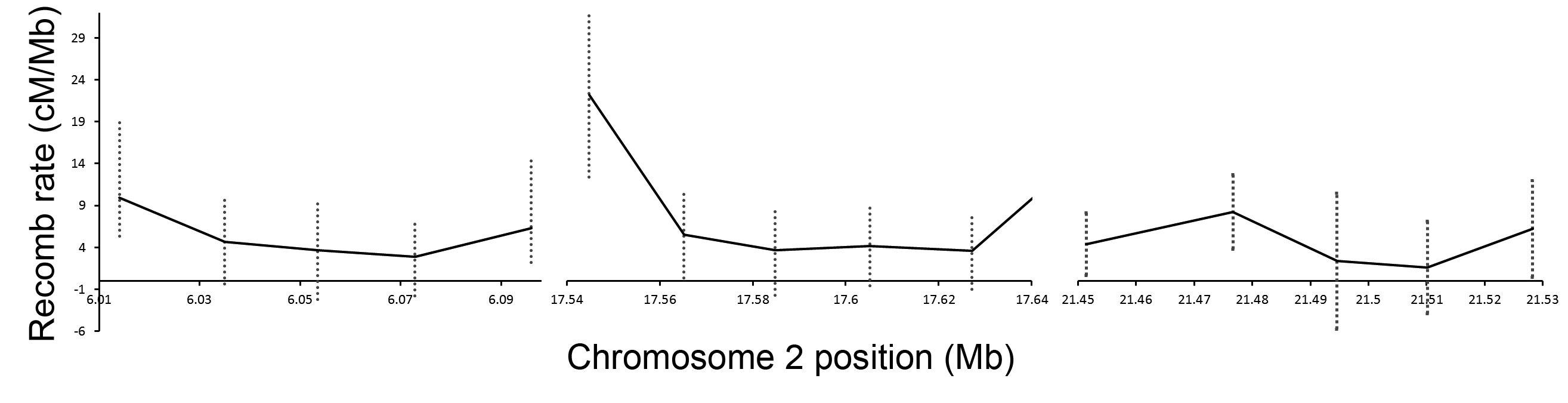

Supplement: Figure S1 — Recombination rate over the 100–125 kb regions. Each window is approximately 20kb. Approximately 10,000 individuals were scored across each window. A similar figure was presented in [17]. (TIF) [file pone.0071582.s001.tif]

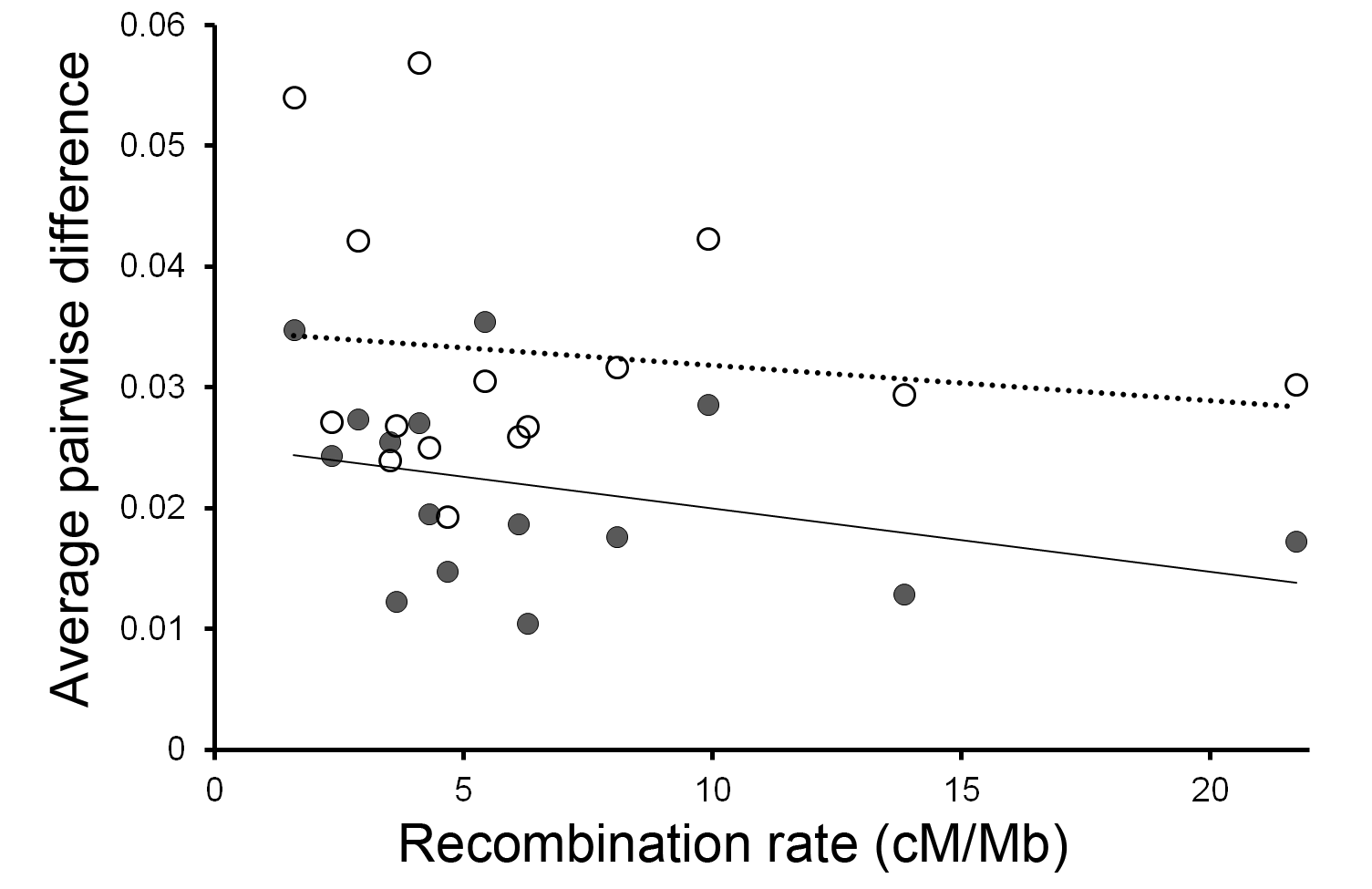

Supplement: Figure S2 — Diversity and divergence in relation to recombination rate for four-fold degenerate sites. Kosambi recombination rate relative to diversity within D. pseudoobscura (grey circles, t = −1.3297, df = 13, p = 0.2067) and divergence between D. pseudoobscura-D. miranda (white circles, t = −0.5098, df = 13, p = 0.6187) for the intervals using four fold degenerate bases in the measure of diversity and divergence. The number of data points was governed by the availability of sites for diversity and divergence measures in each recombination interval; thus, the number of data points in this figure is different from the analogous Figure2 which used data from bases in intergenic regions. Divergence: y = −0.0003x+0.0348, R2 = 0.0196; Diversity: y = −0.0005x+0.0252, R2 = 0.1197. (TIF) [file pone.0071582.s002.tif]
