# Supplementary material for: How Hot Are Drosophila Hotspots? Examining Recombination Rate Variation and Associations with Nucleotide Diversity, Divergence, and Maternal Age in Drosophila pseudoobscura
Source: PLoS One. 2013 Aug 13;8(8):e71582. doi: 10.1371/journal.pone.0071582 (PMC3742509; doi:10.1371/journal.pone.0071582)
Supplement: Table S1 — Test for relationship between recombination rate and diversity and divergence at four-fold degenerate sites. Two generalized linear mixed models with binomial distribution. Region (6 Mb, 17 Mb, 21 Mb) was included as a random effect to account for including multiple intervals per region. This analysis only included 20kb regions. For this analysis, the ‘neutral mutation rate’ was set as the average pairwise D. lowei-D. persimilis divergence at four-fold degenerate sites. (DOCX) [file pone.0071582.s003.docx]

**Table S1.** Test for relationship between recombination rate and diversity and divergence at four-fold degenerate sites. Two generalized linear mixed models with binomial distribution. Region (6Mb, 17Mb, 21Mb) was included as a random effect to account for including multiple intervals per region. This analysis only included 20kb regions. For this analysis, the ‘neutral mutation rate’ was set as the average pairwise *D. lowei*-*D. persimilis* divergence at four-fold degenerate sites.

| **Response: Diversity at four-fold degenerate sites** | | | | | |
| --- | --- | --- | --- | --- | --- |
| **Factor tested** | **Estimate** | **Std. error** | **z-value** | **p-value** |  |
| (Intercept) | -2.657 | 3.325 | -0.799 | 0.424 |  |
| Mutation | 5.301 | 5.139 | 1.032 | 0.302 |  |
| GC content | -2.798 | 7.216 | -0.388 | 0.698 |  |
| Gene density | 0.129 | 0.794 | 0.162 | 0.871 |  |
| **Recombination** | **-0.026** | **0.030** | **-0.870** | **0.384** |  |

| **Response: Divergence at four-fold degenerate sites** | | | | | |
| --- | --- | --- | --- | --- | --- |
| **Factor tested** | **Estimate** | **Std. error** | **z-value** | **p-value** |  |
| (Intercept) | -2.037 | 2.820 | -0.722 | 0.470 |  |
| Mutation | 7.873 | 4.177 | 1.885 | 0.060 |  |
| GC content | -3.246 | 6.135 | -0.529 | 0.597 |  |
| Gene density | -0.434 | 0.689 | -0.631 | 0.528 |  |
| **Recombination** | **-0.024** | **0.0245** | **-0.976** | **0.329** |  |
